# Murine cytomegalovirus infection of mouse macrophages stimulates early expression of suppressor of cytokine signaling (SOCS)1 and SOCS3

**DOI:** 10.1371/journal.pone.0171812

**Published:** 2017-02-09

**Authors:** Christine I. Alston, Richard D. Dix

**Affiliations:** 1 Viral Immunology Center, Department of Biology, Georgia State University, Atlanta, Georgia, United States of America; 2 Department of Ophthalmology, Emory University School of Medicine, Atlanta, Georgia, United States of America; University of St Andrews, UNITED KINGDOM

## Abstract

Human cytomegalovirus (HCMV) is a species-specific β-herpesvirus that infects for life up to 80% of the world’s population and causes severe morbidity in at-risk immunocompromised populations. Suppressors of cytokine signaling (SOCS)1 and SOCS3 are host proteins that act as inducible negative feedback regulators of cytokine signaling and have been implicated in several ocular diseases and viral infections. We recently found in our mouse model of experimental cytomegalovirus retinitis that subretinally-injected murine cytomegalovirus (MCMV) stimulates ocular SOCS1 and SOCS3 during retrovirus-induced immune suppression of murine AIDS (MAIDS), and that infiltrating macrophages are prominent cellular sources of retinal SOCS1 and SOCS3 expression. Herein we investigate possible virologic mechanisms whereby MCMV infection may stimulate SOCS1 and/or SOCS3 expression in cell culture. We report that infection of IC-21 mouse macrophages with MCMV propagated through the salivary glands of BALB/c mice, but not from tissue culture in C57BL/6 fibroblasts, transiently stimulates SOCS1 and SOCS3 mRNA transcripts, but not SOCS5 mRNA. Viral tegument proteins are insufficient for this stimulation, as replication-deficient UV-inactivated MCMV fails to stimulate SOCS1 or SOCS3 in IC-21 macrophages. By contrast, infection of murine embryonic fibroblasts (MEFs) with either productive MCMV or UV-inactivated MCMV significantly stimulates SOCS1 and SOCS3 mRNA expression early after infection. Treatment of MCMV-infected IC-21 mouse macrophages with the antiviral drug ganciclovir significantly decreases MCMV-stimulated SOCS3 expression at 3 days post-infection. These data suggest cell type-specific, different roles for viral immediate early or early gene expression and/or viral tegument proteins in the early stimulation of SOCS1 and SOCS3 during MCMV infection. Furthermore, putative biphasic stimulation of SOCS3 during late MCMV infection of IC-21 mouse macrophages may occur by divergent virologic mechanisms.

## Introduction

Approximately 80% of the world’s population is infected with human cytomegalovirus (HCMV) [1], a species-specific β-herpesvirus [2, 3] which remains latent in its host for life. Although typically asymptomatic, HCMV is nonetheless capable of causing diseases of high morbidity and mortality in immune compromised individuals [2–4]. Patients latently infected with HCMV who develop HIV/AIDS become susceptible to HCMV-related retinitis [5–8], and this remains the leading cause of blindness in AIDS patients not taking or resistant to combination antiretroviral therapy (cART) [9–11]. Although HCMV replication generally can be controlled by lifelong administration of antiviral drugs such as ganciclovir (GCV), these drugs require frequent dosing, potentially cause harmful side-effects, do not eradicate the virus, and merely slow the progression of HCMV-caused ocular or neuronal damage without reversing it [12–16]. HCMV-related disease therefore remains a serious clinical problem worldwide [9–11].

Because the species-specificity of cytomegaloviruses precludes productive infection of HCMV in animal models [17], murine cytomegalovirus (MCMV) has been widely used to investigate mechanisms of cytomegalovirus infection and pathogenesis both in cell culture and mouse models [2]. In our laboratory, we study AIDS-related HCMV retinitis using a clinically relevant small animal model with retrovirus-induced immune suppression that mimics the symptoms and progression of AIDS in mice (MAIDS), eventually rendering them susceptible to experimental MCMV retinitis [18]. We previously found in this model that subretinally-injected MCMV significantly stimulates intraocular suppressors of cytokine signaling (SOCS)1 and SOCS3 [19], host proteins that are inducible negative feedback regulators of cell signaling.

Under normal physiological conditions in host cells, extracellular cytokines recognized by their specific transmembrane receptors on target cell surfaces initiate an intracellular signaling cascade that stimulates the production of dozens of gene products (reviewed in [20–22]), including SOCS family proteins. Although many cell signaling pathways are capable of inducing SOCS [23–26], cytokines signaling via their cognate receptors that activate Janus kinase (JAK)/signal transducer and activator of transcription (STAT) pathways are major transcriptional stimulators of SOCS proteins (reviewed in [27]). Once induced, SOCS family proteins act intracellularly to regulate signaling by JAK/STAT pathways initiated by antiviral interferons (IFN) and other cytokines such as interleukin (IL)-6 [27–31]. In particular, SOCS1 and SOCS3 have been implicated in the pathogeneses of several viral infections (reviewed in [32]), as viral up-regulation of these host proteins may dysregulate host antiviral strategies and thereby assist virally-infected cells in evading immune destruction. In addition, SOCS5 recently has been shown to contribute to Japanese encephalitis virus infection [33], but it remains uninvestigated during MCMV infection.

Macrophages play critical and sometimes contradictory roles during MCMV infection, dependent partly on their differentiation status or reaction to cytokines such as type I and type II interferons [34–39]. It has been demonstrated by others that macrophages infected with MCMV become resistant to IFN-γ-driven activation in a manner partially dependent upon antiviral type I IFN [37, 39], and/or viral inhibition of the promoter assembly for IFN-γ [40]. SOCS family proteins are therefore uniquely positioned to influence this balance in a cell type-dependent and time-dependent manner because they are inducible negative feedback regulators of cytokine signaling pathways that essentially act by reducing the effectiveness of certain secreted cytokines, and they act intracellularly only in those cells expressing them at any given time. Others have demonstrated that MCMV infection in macrophages *in vitro* causes an increase in SOCS1 and SOCS3 mRNA expression levels from 2 to 24 hours after infection [41]. In addition, the importance of macrophages and macrophage progenitor cells for viral dissemination and latency during systemic MCMV infection has been demonstrated [34, 35, 38, 42–44]. It is unclear what virologic mechanism(s) may be involved in the stimulation of SOCS1 and/or SOCS3 in these or other cell types, or how virally-modulated SOCS proteins may contribute to HCMV and/or MCMV infection.

Like all herpesviruses, HCMV and MCMV undergo a temporal, step-wise viral gene expression and replication cascade (reviewed in [2–4]). These enveloped viruses first recognize receptors on the host cell surface, then attach, adsorb, and release tegument proteins into the host cell. Transcription and translation of viral immediate early (IE) genes follows, then early (E) genes, which allow viral DNA synthesis and expression of late (L) genes. Progeny virions are then assembled and egress from the cell. One or more of these virologic events may drive host cell expression of SOCS1 and/or SOCS3 during MCMV infection.

Herein we examined the effect of MCMV infection on SOCS1 and SOCS3 expression in a cell line of mouse macrophages [45] or murine embryonic fibroblasts (MEFs). We tested the hypothesis that MCMV stimulates host SOCS proteins in macrophages in a manner dependent on early steps of the viral replication cycle, and we used two approaches to disrupt this cycle: ultraviolet (UV) inactivation of the virus, and inhibition of viral replication by the antiviral drug GCV. UV inactivation allows viral attachment, adsorption, and release of tegument proteins into the host cell, but it impedes expression of viral genes and viral DNA replication [46]. GCV acts as a guanosine analog [47], preferentially inhibits viral DNA polymerases [48] and therefore viral replication, and allows viral IE and E gene expression without L gene expression [49]. We report that MCMV infection of mouse macrophages or embryonic fibroblasts results in early, transient stimulation of SOCS1 and SOCS3 mRNA transcripts and SOCS-inducing cytokines, with similar temporal patterns between cell types. This stimulation is abrogated by UV inactivation of the virus in IC-21 mouse macrophages, but not MEFs, thus suggesting that cell type-dependent virologic mechanisms underlie early SOCS1 and SOCS3 stimulation. We also found that at 3 days following infection in IC-21 macrophages, MCMV stimulation of SOCS3 mRNA is significantly reduced by GCV treatment. We therefore conclude that one or more viral IE or E genes or tegument-packaged genetic material, but not parental virus tegument proteins, is likely required for early MCMV-related SOCS1 and SOCS3 stimulation in IC-21 mouse macrophages, but not MEFs. Furthermore, our data suggest possible biphasic stimulation of SOCS3 during late MCMV infection of IC-21 cells that is sensitive to GCV antiviral treatment.

## Materials and methods

### Cells and virus

The Smith strain of MCMV was propagated via passage through the salivary glands of adult female BALB/c mice (Harlan Laboratories, USA) as previously described [18, 50, 51]. Experiments were performed with salivary gland-derived MCMV (SG-MCMV) from at least four independent stock preparations. SG-MCMV stock production in animals was conducted with approval of the Georgia State University Institutional Animal Care and Use Committee (IACUC) (Protocol Number A16040) and in compliance with the Association for Research in Vision and Ophthalmology (ARVO) statement for Use of Animals in Ophthalmic and Vision Research. SG-MCMV stocks were quantified by standard plaque assay in C57BL/6 MEFs acquired from the American Type Culture Collection (ATCC, Manassas, VA, No. SCRC-1002). MEFs were grown in Dulbecco’s modified eagle medium (DMEM) supplemented with 15% fetal bovine serum (FBS), 4 mM L-glutamine, 1% penicillin/streptomycin, 0.1 mg/mL gentomicin, and 1.5 g/L sodium bicarbonate.

To determine whether virus stock preparation technique (passage origin) affects SOCS1 or SOCS3 expression, parent SG-MCMV (Smith) was propagated three times sequentially through tissue culture passage (TC-MCMV). Propagation of two variations of TC-MCMV was achieved by inoculating flasks of MEFs of C57BL/6 origin (ATCC No. SCRC-1002) (designated TC-MCMV C57BL/6) or MEFs of BALB/c origin (BALB/3T3, ATCC No. CCL-163, maintained in DMEM with 10% bovine calf serum) (designated TC-MCMV BALB/c). Cells were infected with a low multiplicity of infection (MOI) of 0.001–0.01 PFU/cell of parent SG-MCMV for 3–4 days, then freeze-thawed and clarified, and the supernatants were purified by ultracentrifugation over a sucrose cushion and stored in liquid nitrogen.

IC-21 mouse macrophages (ATCC No. TIB-186) were maintained in RPMI-1640 medium supplemented with 10% FBS, 1% penicillin/streptomycin, and 0.1 mg/mL gentamicin. Unless otherwise indicated, experiments with IC-21 macrophages were performed with IC-21 medium containing 5% FBS, and medium in all experimental wells was refreshed every 24 hrs.

For experiments utilizing UV inactivation of the virus to assess the necessity of viral gene expression and replication on SOCS1 and/or SOCS3 production, a portion of SG-MCMV from the same stock per experiment was exposed to DNA-damaging UV light (UVi-MCMV) [46]. UV inactivation was accomplished by placing approximately 1 mL of the virus stock in an uncovered dish on ice at a 5-cm distance from the germicidal UV light in a laminar flow hood for 1 hr [40]. All UV-inactivated inocula were tested by back-titration to ensure complete inactivation, such that a 0.1-mL sampling failed to produce plaques in MEFs after 2 weeks. To test whether SOCS1 and/or SOCS3 expression is sensitive to antiviral inhibition of SG-MCMV replication later during infection, some monolayers were treated with various concentrations of the antiviral drug GCV (Sigma-Aldrich, St. Louis, MO). At 15 μM, GCV causes a 50% reduction in MCMV progeny virions [47], and IC-21 cells remain >95% viable at concentrations up to and exceeding 60 μM [52]. For these studies, GCV was dissolved in 10% dimethyl sulfoxide (DMSO) at 10X concentrations. These were diluted to indicated final GCV concentrations (0, 15, or 60 μM) in medium containing 5% FBS, such that all monolayers received 1% final concentration of DMSO. Daily-refreshed medium was supplemented with the appropriate concentrations of GCV or DMSO vehicle control per well for each group.

HCMV IE1 and IE2 are encoded by overlapping regions of the HCMV genome and are alternatively spliced into several gene products [53]. For MCMV, the IE1 and IE3 genes are the alternatively-spliced locational [54] and functional homologues of HCMV IE1 and IE2, respectively [55]. MCMV IE2 has no sequential or functional homologue in the HCMV genome and is dispensable for growth *in vitro* [56] and *in vivo* [57]. MCMV mutant RM4503 [58, 59], from the wild type K181^+^ parent strain, expresses enhanced green fluorescent protein (EGFP) under the control of a fragment of the HCMV promoter-enhancer adjacent to the MCMV IE2 enhancer in the MCMV genome. This mutant was previously constructed by others [58] and was a gift from the laboratory of Dr. Tim Sparer, Department of Microbiology, University of Tennessee, Knoxville. MCMV RM4503 contains the EGFP construct inserted into the MCMV genome to disrupt the MCMV IE2 gene, and it therefore does not express IE2 [58]. Instead, EGFP is expressed with IE2 kinetics [58–60]. Before use in experiments, this tissue culture-passaged mutant virus was propagated three times sequentially through the salivary glands of female BALB/c mice as described for MCMV Smith strain. EGFP expression was detected in live cells infected with MCMV RM4503 under a Nikon Eclipse fluorescent microscope.

### RNA extraction and real-time reverse transcriptase polyacrylamide chain reaction (RT-PCR)

Cell monolayers experimentally treated as specified were harvested at indicated time points in TRIzol^®^ reagent (Ambion/ThermoFisher Scientific, Waltham, MA, USA). Total RNA was isolated by chloroform extraction and purified over PureLink^®^ RNA Mini Kit spin cartridge filters according to the manufacturer’s instructions (Ambion/ThermoFisher). RNA was reverse-transcribed (RT) into cDNA with SuperScript^™^ III First-Strand Synthesis Kit reagents using random hexamers per the manufacturer’s instructions (Invitrogen/ThermoFisher). Real-time RT-PCR was performed using Applied Biosystems 7500 Fast Real-Time PCR System hardware and software with Power SYBR Green Master mix (Applied Biosystems, Foster City, CA) and with primer sets of mouse-specific SOCS1, SOCS3, SOCS5, IFN-α, IFN-β, IFN-γ, IL-6, and glyceraldehyde 3-phosphate dehydrogenase (GAPDH) obtained from QIAgen (Valencia, CA). MCMV IE1 primer sequences (forward: 5'-TCA GCC ATC AAC TCT GCT ACC AAC-3', reverse: 5'-ATC TGA AAC AGC CGT ATA TCA TCT TG-3') were purchased from Integrated DNA Technologies (IDT, Redwood City, CA). Samples were run under the following thermocycling parameters: 10 min at 95°C, followed by 40 cycles consisting of 15 s at 94°C, 31 s at 55°C, and 35 s at 70°C. Cycles to threshold (C_T_) for each target gene were determined, and each sample was normalized to its own endogenous housekeeping gene (GAPDH) by ΔC_T_ analysis (ΔC_T_ = C_T target gene_−C_T GAPDH_). To determine gene expression changes in host cell-derived transcripts, ΔC_T_ values of target gene mRNA in experimental wells were compared with control wells by the 2^-ΔΔCt^ method, yielding a relative fold change in mRNA expression for each treatment group. For expression of viral mRNA, where fold change relative to an uninfected control was inapplicable, MCMV IE1 mRNA was normalized to cellular GAPDH and shown as 2^-ΔCt^ [61]. Unless otherwise stated, all time-course studies for cellular gene expression were analyzed by comparing each sample back to the media control group at 0 hour post-infection (hpi). Data points represent mean fold changes ± standard deviations (SD) of at least duplicate experimental repeats.

### Immunofluorescent (IF) staining

IF staining was performed to test whether MCMV or UV-inactivated MCMV stimulates detectable SOCS1 and SOCS3 protein in macrophages. IC-21 mouse macrophages were grown on German glass cover slips (Electron Microscopy Sciences, Hatfield, PA) in 24-well dishes and infected with 0.5 mL per well of SG-MCMV (MOI = 3 PFU/cell), UVi-MCMV from the same stock, or control medium. At 3 hpi, cover slides from each group were fixed in ice-cold methanol, blocked in 5% bovine serum albumin, and probed for rabbit-anti-mouse SOCS1 or SOCS3 primary antibodies (Santa Cruz Biotechnology, Inc., Dallas, TX). Goat-anti-rabbit IgG Fab’ fragment antibody conjugated with FITC (green) (Jackson ImmunoResearch, West Grove, PA) was used for secondary antibody. Nuclei were counterstained with 4',6-diamidino-2-phenylindole (DAPI) in Vectashield mounting solution (Vector Laboratories, Burlingame, CA), and cover slides fixed to microscope slides were observed under a Nikon Eclipse fluorescent microscope.

IF staining for SOCS1 or SOCS3 was also performed on IC-21 monolayers infected with EGFP-expressing MCMV RM4503 at 3 hpi. SG-MCMV Smith strain served as a positive control for MCMV-stimulated SOCS1 and SOCS3 expression, with media treatment as the negative control. Because EGFP-expressing MCMV RM4503 was viewed in the green channel for this experiment, SOCS1 or SOCS3 primary antibodies were detected in the red channel using goat-anti-rabbit IgG Fab’ fragment secondary antibody conjugated with Cy3 (Jackson ImmunoResearch).

### Statistical analyses

Statistical analyses were performed using GraphPad Prism^®^ v6.07 software with a significance level (α) set to 0.05, so that p-values of <0.05 were considered statistically significant. For Figures, asterisks are used to denote statistical significance as: * p<0.05, ** p<0.01, and *** p<0.001, for experimental groups compared with respective control groups at the same time points by one-way or two-way analysis of variance (ANOVA) where appropriate with Tukey’s post-hoc analysis (time course experiments, passage origin study) or Dunnett’s multiple comparisons test (GCV experiment).

## Results

### SOCS1 and SOCS3 mRNA transcripts are transiently stimulated upon MCMV infection of IC-21 mouse macrophages

**Fig 1:**
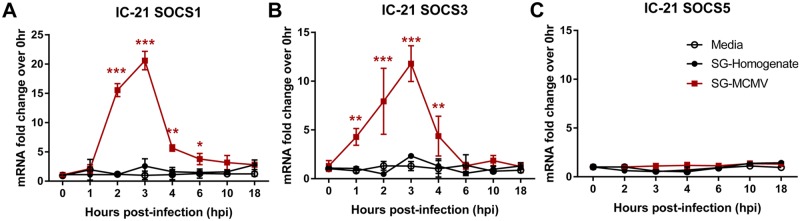
Infection of mouse macrophages with MCMV stimulates SOCS1 and SOCS3, but not SOCS5, mRNA transcripts at early time points. IC-21 mouse macrophages were treated with media (control) or infected with SG-MCMV (MOI = 3 PFU/cell) or SG-homogenate. At indicated time points, cells were harvested and assessed for SOCS1 mRNA **(A)**, SOCS3 mRNA **(B)**, or SOCS5 mRNA **(C)** by real-time RT-PCR assay using the comparative 2^-ΔΔCt^ method, with all samples compared back to the media group at 0 hpi. Means ±SD of triplicate experimental repeats are shown. * p<0.05, ** p<0.01, and *** p<0.001, compared with respective media controls at the same time points.

Macrophages are important participants during MCMV infection [34–39]. Others have demonstrated that infection of bone marrow-derived macrophages with MCMV passaged through BALB/c-derived MEFs causes an increase in SOCS1 and SOCS3 mRNA expression levels from 2 to 24 hours after infection [41]. To our knowledge, it was unknown whether MCMV propagated through the salivary glands of BALB/c mice (SG-MCMV) stimulates SOCS1, SOCS3, and/or SOCS5 expression in the IC-21 cell line of C57BL/6-derived mouse macrophages. We found that SOCS1 (Fig 1A) and SOCS3 (Fig 1B), but not SOCS5 (Fig 1C), mRNA transcripts are up-regulated at early time points following infection of IC-21 monolayers with SG-MCMV. Treatment of IC-21 monolayers with BALB/c mouse salivary gland homogenate (SG-homogenate) failed to stimulate SOCS1, SOCS3, or SOCS5 mRNA transcripts during the same time points (Fig 1). Peak SOCS1 and SOCS3 mRNA stimulation occurred between 2–6 hpi, a relatively early time during productive MCMV infection that follows viral attachment, adsorption, and release of viral tegument proteins into the host cell and correlates with transcription and translation of MCMV IE genes in fibroblast cells [62, 63] (reviewed in [2–4]). Because SOCS5 mRNA expression was not stimulated during SG-MCMV infection at any time points observed, this gene target was not examined in subsequent experiments.

### MCMV-related SOCS1 and SOCS3 stimulation in IC-21 mouse macrophages is dependent on virus passage origin (SG-MCMV or TC-MCMV)

**Fig 2:**
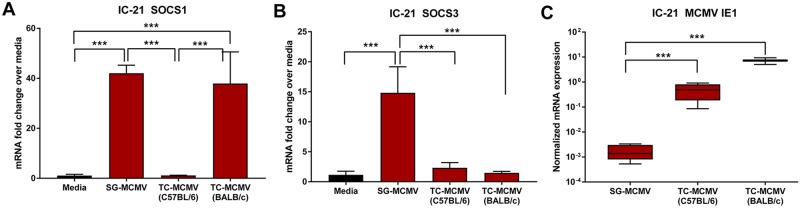
MCMV-related SOCS1 and SOCS3 stimulation in macrophages at 3 hpi is dependent on viral passage origin (SG-MCMV or TC-MCMV). IC-21 mouse macrophages were treated with media (baseline control) or infected with SG-MCMV (from BALB/c salivary glands), TC-MCMV from C57BL/6 MEFs, or TC-MCMV from BALB/c MEFs at MOI = 3 PFU/cell. At 3 hpi, cells were harvested and assessed for SOCS1 **(A)** or SOCS3 **(B)** mRNA by real-time RT-PCR assay by the comparative 2^-ΔΔCt^ method, with all groups relative to the media control group. Means ±SD of at least duplicate experimental repeats are shown. Expression of MCMV IE1 mRNA **(C)** of the virus-infected groups was normalized to GAPDH transcripts and analyzed by the comparative 2^-ΔCt^ method. Minimum and maximum IE1 mRNA expression values (red boxes) and means (black lines within boxes) ±SD of at least duplicate experimental repeats are shown on a logarithmic scale. *** p<0.001 between bracketed groups by one-way ANOVA with Tukey’s post-hoc analysis.

Because SG-MCMV and TC-MCMV display many virologic, immunologic, and pathologic differences *in vitro* and *in vivo* [64–67], particularly in relation to macrophage infection [64–69], we tested whether MCMV-related stimulation of SOCS1 and/or SOCS3 is affected by viral passage origin (SG-MCMV or TC-MCMV), and, more specifically, the mouse strain (C57BL/6 or BALB/c) of the host MEFs for TC-MCMV propagation. We found in monolayers of IC-21 mouse macrophages that SG-MCMV, but not TC-MCMV from C57BL/6 MEFs, highly stimulated SOCS1 and SOCS3 mRNA transcripts at 3 hpi (Fig 2). Moreover, TC-MCMV from BALB/c MEFs up-regulated SOCS1, but not SOCS3, at this time point.

### UV-inactivated MCMV does not significantly stimulate SOCS1 or SOCS3 expression in IC-21 mouse macrophages

**Fig 3:**
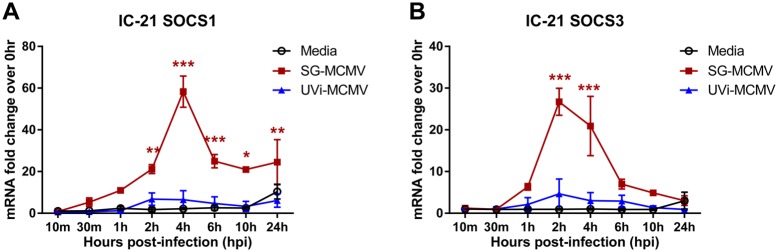
UV-inactivation of MCMV abrogates MCMV-related stimulation of SOCS1 and SOCS3 mRNA transcripts in mouse macrophages. IC-21 mouse macrophages treated with media (control), infected with SG-MCMV (MOI = 3 PFU/cell), or treated with an equal volume of UVi-MCMV were assessed for SOCS1 mRNA **(A)** or SOCS3 mRNA **(B)** by real-time RT-PCR assay using the comparative 2^-ΔΔCt^ method, with all samples compared back to the media group at 0 hpi. Means ±SD of at least duplicate experimental repeats are shown. * p<0.05, ** p<0.01, and *** p<0.001, compared with respective media controls at the same time points. No statistically significant differences were found between the media controls and the UV-MCMV groups at any time point.

**Fig 4:**
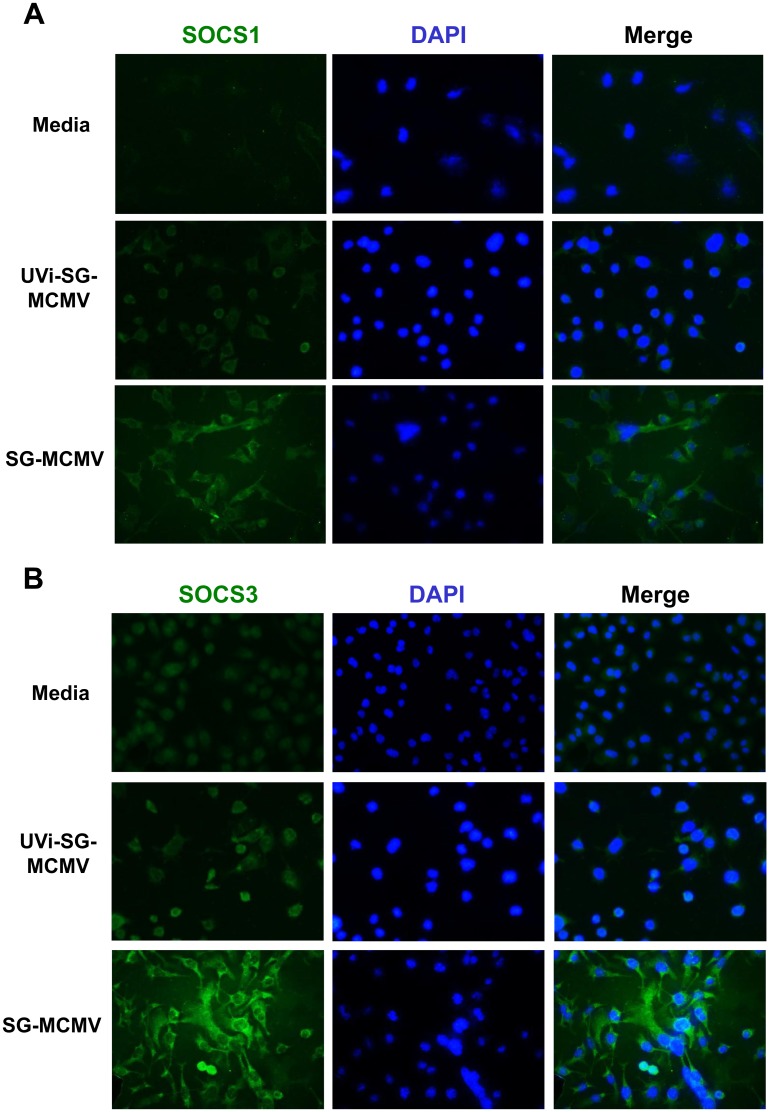
UV-inactivation of MCMV reduces MCMV-related stimulation of SOCS1 and SOCS3 protein in mouse macrophages. IC-21 mouse macrophages grown on glass cover slides were treated with media (control), infected with SG-MCMV (MOI = 3 PFU/cell), or treated with an equal volume of UVi-MCMV. All groups were fixed at 3 hpi and assessed by immunofluorescent staining for SOCS1 **(A)** or SOCS3 protein **(B)** (green), counterstained with DAPI (blue). Original magnification, 400x.

To test the hypothesis that the early virologic events of attachment, adsorption, and release of tegument proteins are sufficient for SOCS1 and SOCS3 mRNA stimulation during MCMV infection, we infected monolayers of IC-21 macrophages or MEFs with SG-MCMV exposed to DNA-damaging UV light (UVi-MCMV). This technique leaves cellular or immunologic components of MCMV intact while rendering the virus deficient in viral gene expression and replication, thereby allowing attachment, adsorption, and release of tegument proteins into the infected cell [46]. In contrast with the significant stimulation of SOCS1 and SOCS3 mRNA transcripts in IC-21 mouse macrophages during productive SG-MCMV infection, UVi-MCMV resulted in only a small trend toward transient stimulation of SOCS1 and SOCS3 that did not reach statistical significance when compared with medium-treated control cells (Fig 3) and remained significantly lower than SG-MCMV values (SOCS1: p<0.05 for ≥2 hpi, and SOCS3: p<0.001 for 2 and 4 hpi, UVi-MCMV compared with SG-MCMV at each time point). In IC-21 cells immunofluorescently labeled with anti-SOCS1 or anti-SOCS3 antibodies, treatment with medium or UVi-MCMV resulted in basal to moderate SOCS1 or SOCS3 protein expression, while SG-MCMV infection (MOI = 3 PFU/cell, 3 hpi) caused robust stimulation of these proteins (Fig 4), found mostly in the cytoplasm.

**Fig 5:**
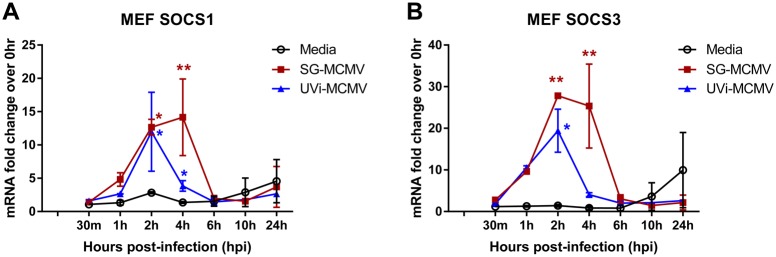
MCMV infection stimulates mRNA expression of SOCS1 and SOCS3 mRNA in MEFs. MEFs treated with media (control), infected with SG-MCMV (MOI = 3 PFU/cell), or treated with an equal volume of UVi-MCMV were assessed at indicated time points by real-time RT-PCR assay using the comparative 2^-ΔΔCt^ method for SOCS1 **(A)** or SOCS3 **(B)** mRNA transcripts, with samples compared back to the media group at 0 hpi. Means ±SD of at least duplicate experimental repeats are shown. * p<0.05 and ** p<0.01, compared with media controls at the same time points.

Productive SG-MCMV infection of MEFs produced similar temporal patterns of SOCS1 and SOCS3 mRNA expression to those found in IC-21 cells (Fig 5). Unlike UVi-MCMV infection of IC-21 cells, which produced no significant up-regulation of SOCS1 or SOCS3 transcripts during the time points observed in these macrophages, UVi-MCMV infection of MEFs caused significant, albeit transient, stimulation of SOCS1 (Fig 5A) and SOCS3 (Fig 5B) mRNA expression at 2 hpi.

### Cytokines known to induce expression of SOCS1 and SOCS3 are up-regulated during MCMV infection of IC-21 and MEFs

**Fig 6:**
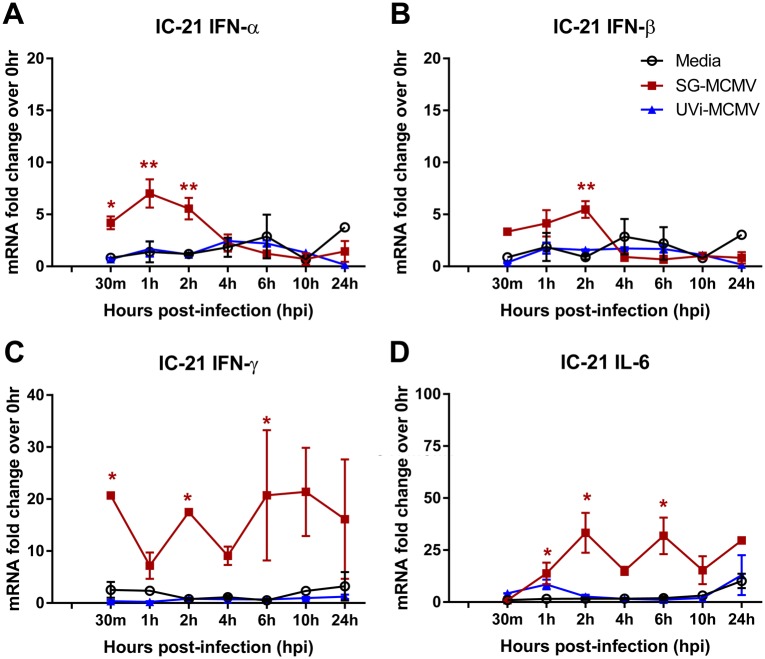
Infection of mouse macrophages with SG-MCMV, but not UV-inactivated SG-MCMV, stimulates cytokines known to induce SOCS1 and SOCS3. Monolayers of IC-21 mouse macrophages were treated with media (control), infected with MCMV at MOI = 3 PFU/cell, or treated with an equal volume of UVi-MCMV. Cells were harvested at indicated time points and assessed by real-time RT-PCR assay using the comparative 2^-ΔΔCt^ method for IFN-α **(A)**, IFN-β **(B)**, IFN-γ **(C)**, or IL-6 **(D)** mRNA transcripts, with all samples compared back to the media group at 0 hpi. Means ±SD of at least duplicate experimental repeats are shown. * p<0.05, ** p<0.01, and *** p<0.001, compared with respective media controls at the same time points.

Because SOCS transcripts are readily induced by cytokine signaling through the JAK/STAT pathway [27, 28, 70, 71], and MCMV infection causes up-regulation of many cytokines such as IFN-γ [72, 73] and IL-6 [74], we next asked whether such cytokines are concurrently stimulated with SOCS1 and SOCS3 mRNA transcripts during SG-MCMV infection of IC-21 mouse macrophages or MEFs. We reasoned that if MCMV stimulation of SOCS1 and SOCS3 transcripts is a consequence of viral stimulation of SOCS-inducing cytokines, then transcripts for these cytokines would also be up-regulated, likely preceding stimulation of the SOCS transcripts. SG-MCMV infection of IC-21 mouse macrophages resulted in transient stimulation of mRNA transcripts for antiviral type I IFNs (IFN-α and IFN-β, Fig 6A and 6B, respectively) in agreement with previous findings for TC-MCMV infection by others [75], and prolonged stimulation of type II IFN (IFN-γ) (Fig 6C) and IL-6 (Fig 6D) mRNA. For all time points observed, infection of IC-21 cells with UVi-MCMV failed to stimulate these cytokines beyond the levels of the medium-treated control wells (Fig 6).

**Fig 7:**
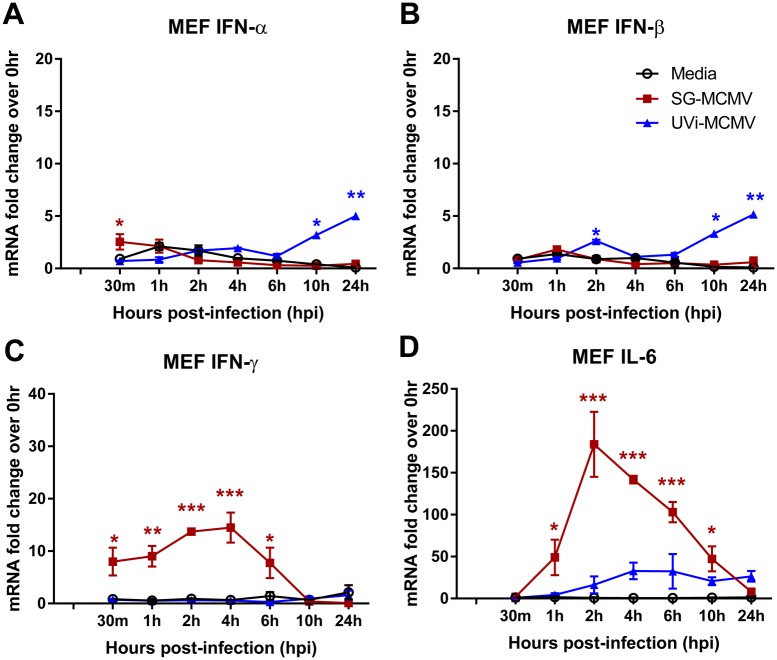
SOCS-inducing cytokines IFN-α, IFN-γ, and IL-6, but not IFN-β, are transcriptionally stimulated during MCMV infection of MEFs. MEFs treated as in Fig 5 with media (control), MCMV (MOI = 3 PFU/cell), or an equal volume of UVi-MCMV were assessed at indicated time points by real-time RT-PCR assay using the comparative 2^-ΔΔCt^ method for IFN-α **(A)**, IFN-β **(B)**, IFN-γ **(C)**, or IL-6 **(D)** mRNA transcripts, with all samples compared back to the media group at 0 hpi. Means ±SD of at least duplicate experimental repeats are shown. * p<0.05, ** p<0.01, and *** p<0.001, compared with media controls at the same time points.

In MEFs, MCMV infection resulted in moderate, early stimulation of IFN-α mRNA transcripts (Fig 7A), but not IFN-β mRNA transcripts (Fig 7B), at 30 min post-infection, with subsequent dampening of these type I IFNs beyond 30 min. Infection with UVi-MCMV caused significant up-regulation of these type I IFNs at later time points (10, 24 hpi). Transcripts of IFN-γ (Fig 7C) and IL-6 (Fig 7D) mRNA were highly stimulated in MEFs following productive MCMV infection, but not UVi-MCMV.

### EGFP-tagged SG-MCMV RM4503 stimulates SOCS1 and SOCS3 protein expression in IC-21 macrophages prior to detection of IE2 promoter-driven EGFP

**Fig 8:**
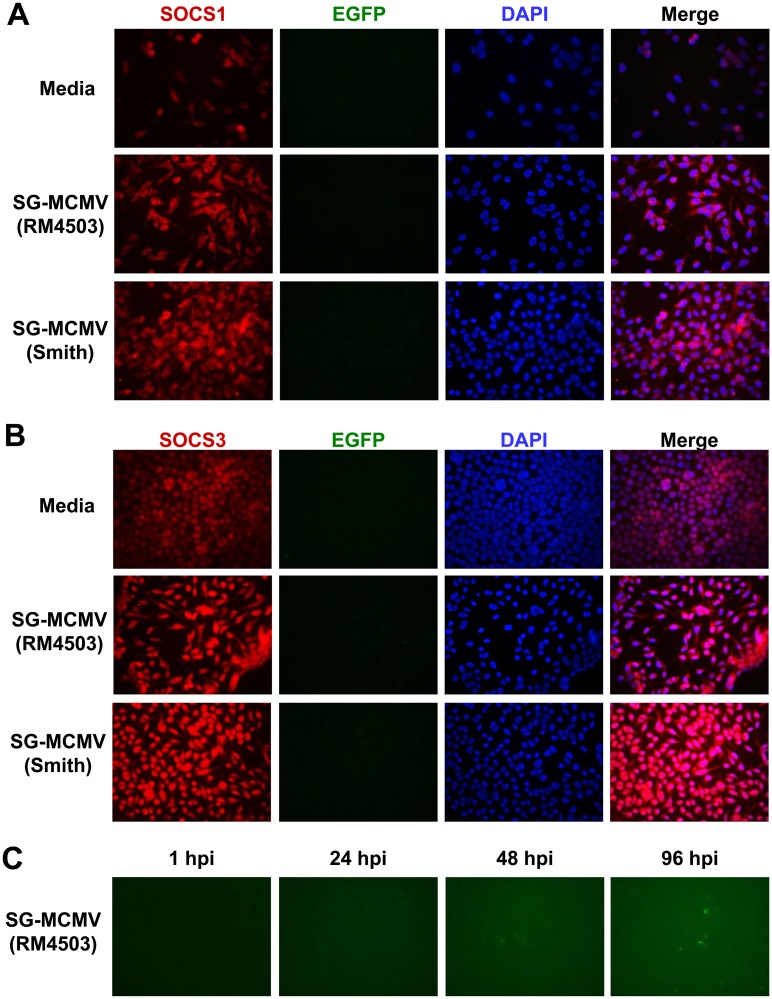
Infection of IC-21 mouse macrophages with EGFP-expressing SG-MCMV RM4503 stimulates SOCS1 and SOCS3 proteins, despite lack of detection of EGFP. IC-21 cells were treated with media or infected (MOI = 3 PFU/cell) with wild type SG-MCMV (Smith) or SG-MCMV mutant RM4503, which expresses EGFP under the control of the IE2 promoter. At 3 hpi, cells were methanol-fixed and stained with antibodies detecting SOCS1 **(A)** or SOCS3 **(B)** (red). SOCS1 and SOCS3 expression was increased, despite lack of detection of MCMV IE2-driven EGFP (green) in these cells at 3 hpi. Original magnification 400×. **(C)** In live IC-21 macrophages infected with SG-MCMV mutant RM4503, IE2 promoter-driven EGFP was not visibly detected until 48 hpi and increased thereafter. Original magnification 200×.

Because MCMV infection potently stimulates transcription of host cell proteins [4], including SOCS-inducing cytokines [72–74], SOCS1 and/or SOCS3 stimulation may occur, in whole or in part, by an indirect effect on uninfected bystander cells. To test for this possibility, we infected IC-21 macrophages with the EGFP-expressing tracer virus MCMV RM4503 [58, 60] and assessed whether EGFP expression co-localizes with immunofluorescently-stained SOCS1 or SOCS3 proteins. SOCS1 and SOCS3 proteins were stimulated in IC-21 cells at 3 hpi by wild type SG-MCMV (Smith) or by SG-MCMV RM4503 compared with baseline expression in media-treated control cells (Fig 8A and 8B). At this time point, however, IE2 promoter-driven EGFP was not detected in IC-21 cells infected with SG-MCMV RM4503, despite expected amounts of infectious virus and plaque-associated EGFP expression upon back-titration of the inoculum in MEFs.

MCMV RM4503 expresses EGFP with IE2 kinetics, and green fluorescence is detectable at 6 hpi in infected NIH/3T3 fibroblasts [58]. We therefore expected to find IE2 promoter-driven EGFP expression very early during infection in IC-21 cells [76], but EGFP was undetected at 3 hpi. To determine the expression kinetics of IE2 promoter-driven EGFP in these cells, we infected monolayers of IC-21 mouse macrophages with MCMV RM4503 and periodically screened the live cells for EGFP expression under a fluorescent microscope. We did not visually detect EGFP from within MCMV RM4503-infected IC-21 cells until 48 hpi (Fig 8C).

### Ganciclovir (GCV) treatment decreases MCMV-stimulated SOCS3 production in IC-21 mouse macrophages

**Fig 9:**
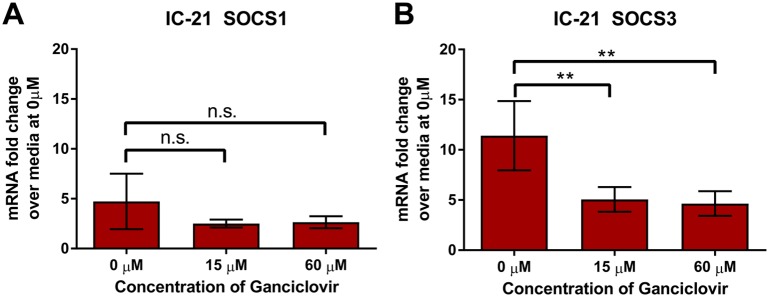
Ganciclovir (GCV) treatment of MCMV-infected mouse macrophages reduces MCMV-related stimulation of SOCS3 mRNA at 72 hpi. IC-21 mouse macrophages were treated with media (baseline control) or infected with SG-MCMV (MOI = 3 PFU/cell). At 1 hpi, wells were treated with the antiviral drug GCV at the indicated final concentrations, or vehicle control (0 μM). At 72 hpi, cells were harvested and assessed for SOCS1 **(A)** or SOCS3 **(B)** mRNA by real-time RT-PCR assay using the comparative 2^-ΔΔCt^ method, with all samples compared back to the vehicle-treated (0 μM) media group. Means ±SD of at least triplicate experimental repeats are shown. * p<0.05, ** p<0.01, and n.s.: not significant, for MCMV-infected GCV-treated groups compared with MCMV-infected vehicle controls.

Because UV-inactivated MCMV failed significantly to stimulate SOCS1 and SOCS3 expression at early time points following infection of IC-21 mouse macrophages, we next investigated SOCS1 and SOCS3 mRNA expression later during MCMV infection (72 hpi) with or without the antiviral drug GCV. This drug has been shown to inhibit MCMV replication and subsequent expression of late viral genes [49] with a 50% inhibitory concentration (IC_50_) of 15 μM *in vitro* [47]. At 72 hrs following MCMV infection of IC-21 monolayers, GCV treatment significantly reduced MCMV-stimulated SOCS3 mRNA transcripts (Fig 9B), with SOCS1 mRNA expression displaying only a downward trend with GCV treatment that did not reach statistical significance (Fig 9A).

## Discussion

Herein we characterized the expression of two prominent members of the SOCS family, SOCS1 and SOCS3, during SG-MCMV infection in cell culture models. We also investigated the heretofore unknown effect of MCMV infection on SOCS5 production in macrophages. Although SOCS5 has been shown by others to play a role during Japanese encephalitis virus infection [33], we did not observe SOCS5 stimulation during MCMV infection of IC-21 mouse macrophages, thus underscoring the specificity of individual viruses to affect their hosts diversely and uniquely. We report the new finding that MCMV-related early stimulation of SOCS1 and SOCS3 mRNA in macrophages is affected by viral passage origin (SG-MCMV or TC-MCMV) and, more specifically, the mouse strain of the host cells used for TC-MCMV propagation. We looked at various time points following SG-MCMV infection and used two antiviral techniques targeting different events in the viral replication cycle to test the hypothesis that MCMV-related SOCS1 and/or SOCS3 production is dependent upon early steps of this cycle. The results of this study demonstrate that expression of one or more viral IE or E genes, or tegument-packaged non-coding RNA, is likely required for early MCMV-related SOCS1 and SOCS3 stimulation in IC-21 mouse macrophages, but not in MEFs. By contrast, tegument proteins from parental virus may play a role in SOCS1 and/or SOCS3 stimulation during MCMV infection of MEFs, but not IC-21 macrophages. These data therefore suggest that one or more cell type-dependent virologic mechanisms underlie early SOCS1 and SOCS3 stimulation during MCMV infection. Furthermore, biphasic stimulation of SOCS3 during early and then late MCMV infection of IC-21 cells may occur by temporally divergent virologic and/or immunologic mechanisms.

Infection of IC-21 mouse macrophages or MEFs with SG-MCMV resulted in early, transient stimulation of SOCS1 and SOCS3 mRNA expression compared with medium-treated controls. SOCS1 or SOCS3 stimulation in IC-21 mouse macrophages could not be attributed to immune or antigen factors in the SG-homogenates of uninfected mice, nor from any soluble factors found in SG-MCMV stocks rendered noninfectious by UV inactivation. In agreement with these mRNA data, immunofluorescent staining of IC-21 macrophages for SOCS1 or SOCS3 revealed robust stimulation of these proteins at 3 hrs following infection with productive MCMV, but not after treatment with UVi-MCMV or media. Furthermore, SOCS1 and SOCS3 proteins following MCMV infection appeared mostly in the cytoplasm, where these proteins undergo their major suppressive functions (reviewed in [25]).

Although peak SOCS1 and SOCS3 mRNA expression occurred at a time when expression of viral IE and E proteins during MCMV infection of fibroblasts has been reported [62, 63], it cannot be ignored that MCMV gene expression kinetics in macrophages are different from those that have been reported for fibroblasts. Others have previously shown that TC-MCMV replication curves and gene expression kinetics are delayed in macrophages compared with fibroblasts [35, 38, 75]. The current study adds similar results for SG-MCMV infection in that MCMV IE1 mRNA transcripts in IC-21 mouse macrophages are reduced or delayed until 48 hpi or later compared with IE1 expression in MEFs. We also found that expression of the IE2 promoter-driven EGFP of SG-MCMV RM4503 was not visually detectable in IC-21 cells until 48 hpi (Fig 8C). Others have investigated the temporal kinetics of MCMV IE, E, and L gene mRNA transcripts following infection of tissue culture-passaged MCMV (TC-MCMV) BAC-derived strain MW97.01 parent virus in IC-21 mouse macrophages and showed significant mRNA expression of the IE2 gene (m128) as early as 1 hpi in these cells [76]. Possible explanations for this discrepancy in IE kinetics during MCMV infection of IC-21 macrophages may arise from the use of different viral strains or different passage sources of the MCMV stocks.

Others have shown SOCS1 and SOCS3 transcriptional stimulation following TC-MCMV at 2, 8, and 24 hpi in bone marrow-derived macrophages from C57BL/6 mice [41]. The TC-MCMV stock from their study was propagated through BALB/c MEFs, and we confirmed that SOCS1, albeit not SOCS3 at this time point, is stimulated at 3 hpi by TC-MCMV from BALB/c MEFs. Therefore, MCMV-stimulated expression of SOCS1, and perhaps SOCS3, is dependent not only on virus passage origin, but more specifically on the mouse strain of the host cells used for propagation of the MCMV stocks. Although this begs the question of host cell residue antigenicity in the viral stock preparations, this is unlikely because: (i) uninfected salivary gland homogenate from BALB/c mice failed to stimulate SOCS1 and SOCS3 in IC-21 cells (Fig 1), (ii) UV-inactivated SG-MCMV, which should contain all antigens present in the stocks of productive SG-MCMV, failed to stimulate SOCS1 and SOCS3 in IC-21 cells, and (iii) the TC-MCMV from BALB/c MEFs in the current study was purified by ultracentrifugation, and the virus used by others (41) was filtered through a 0.45-um pore, techniques which would presumably eliminate most host tissue residue from the stock.

The precise mechanism(s) by which MCMV virions of different passage origins enforce their divergent effects on SOCS1 expression remains to be seen and is the topic of an ongoing project in our laboratory. Attenuation by viral gene mutation through serial passage in C57BL/6, but not BALB/c, MEFs is possible, however unlikely. This possibility is consistent with the well-established observation that MCMV is rapidly attenuated after cell culture passage, but virulence is restored after subsequent salivary gland passages [68, 77]. Such a situation would further suggest that the mechanism driving some or all phenotypic differences between SG-MCMV and TC-MCMV passage origins is not due to genotypic differences [68, 77, 78] but may be found on a proteomic level. Comparative genomic or proteomic analyses could be employed in future studies to determine whether this is the case, or whether a role exists for the different major histocompatibility H-2 haplotypes between mouse strains. Another intriguing explanation for this difference stems from the finding that HCMV and MCMV readily package host proteins [79] and host RNA [80] into their teguments. This possibility could allow for a SOCS-inducing host-derived factor that SG-MCMV, or TC-MCMV from BALB/c MEFs, packages into the tegument but that is not present in cell cultures used for TC-MCMV stocks from C57BL/6 MEFs. Whatever the mechanism, it is clear that the ability of MCMV infection to stimulate at least SOCS1 expression in macrophages is affected by the host cell origin of the virions.

Attachment, adsorption, and release of viral tegument proteins in the absence of viral gene expression (UVi-MCMV) were insufficient to cause significant stimulation of SOCS1 or SOCS3 in IC-21 mouse macrophages (Fig 3), but were sufficient to stimulate these transcripts in MEFs (Fig 5). These data suggest a cell type-specific putative role for one or more IE or E protein(s), but not parental virus tegument proteins, in the expression of SOCS1 and SOCS3 during MCMV infection of mouse macrophages. Because host or viral RNA or other genetic material packaged into the tegument would also be expected to be damaged during UV inactivation, we therefore have not yet eliminated the possibility that infection of IC-21 cells may stimulate SOCS1 and/or SOCS3 by one or more viral or host transcripts that are packaged into the tegument of SG-MCMV.

It is clear from the results of this study that, at least at a single time point, IE1 expression is not a likely contributor to the stimulation of SOCS1 and SOCS3 in IC-21 macrophages because SG-MCMV stimulated the greatest amounts of SOCS1 and SOCS3 while concurrently producing the lowest amounts of IE1 (Fig 2). As we have not ruled out a threshold effect or earlier stimulation of IE1 not seen at this time point, more extensive time course studies with IE1 knockout or suppression are necessary to confirm what role, if any, IE1 has on the direct or indirect stimulation of SOCS1 and/or SOCS3. Furthermore, our experiment with MCMV RM4503 revealed the dispensability of IE2 for the stimulation of SOCS1 and SOCS3 in macrophages. MCMV RM4503 contains the EGFP gene inserted into the MCMV genome to disrupt the MCMV IE2 gene, and it therefore does not express IE2 [58]. Instead, EGFP is expressed with IE2 kinetics [58–60]. Therefore, MCMV-related stimulation of SOCS1 and SOCS3 occurred during infection of IC-21 mouse macrophages with the IE2-defective MCMV RM4503 [58, 59], suggesting that IE2 is dispensable for this phenotype. Although the physiologic significance and kinetics of IE and/or E genes at other time points under the parameters of this study remain unclear, neither expression levels alone of IE1 transcripts, nor the presence of IE2, is sufficient to drive SOCS1 or SOCS3 production in macrophages. Therefore, the effect(s) of IE gene expression, particularly that of IE3, as well as E gene products on SOCS1 and SOCS3 production in IC-21 macrophages requires further investigation.

In IC-21 mouse macrophages, transcripts for the SOCS-inducing cytokines antiviral type I IFNs (IFN-α and IFN-β), type II IFN (IFN-γ), and IL-6 were significantly up-regulated during productive SG-MCMV infection over media-treated controls at time points corresponding with or preceding SOCS1 and SOCS3 up-regulation, and the same was true in MEFs with the exception of IFN-β. The transient, early stimulation of type I IFN in IC-21 mouse macrophages and fibroblasts is consistent with previous findings by others [75] for TC-MCMV infection of these cells at high MOIs (5 and 15 PFU/cell for fibroblasts and IC-21 cells, respectively). Given these data, we cannot ignore the possibility that stimulation of all or one of these cytokines could play a role in SOCS1 and SOCS3 up-regulation during MCMV infection of these cells. Such a role for type I IFNs may seem unlikely because TC-MCMV contains several cell type-specific mechanisms to inhibit various type I IFN-inducing pathways [75], but to our knowledge, it remains unclear whether SG-MCMV does the same.

As with SOCS1 and SOCS3 transcripts in IC-21 cells, viral gene expression was necessary for stimulation of type I and II IFN and IL-6 because UVi-MCMV failed to stimulate any of these SOCS-inducing cytokines at any time point examined, lending further support to the possibility that these cytokines may contribute to SOCS expression during productive MCMV infection. The unexpected up-regulation of type I IFN mRNA transcripts in MEFs by UVi-MCMV, but not productive MCMV infection, at later time points (10, 24 hpi) might be explained by the presence of a cell type-specific, virally-encoded inhibitor of type I IFN transcription, as has been observed by others [75]. These data suggest that the virologic mechanisms for stimulation of SOCS-inducing cytokines are different depending on cell type, and the possibility cannot be excluded that SOCS1 and SOCS3 expression may be stimulated, in part or whole, as an indirect immunologic consequence of MCMV infection stimulating these or other cytokines.

SOCS1 and SOCS3 can be transcriptionally up-regulated by activation of the JAK/STAT pathway [27, 28, 70, 71], and because tyrosine phosphorylation of STAT proteins is required for their transcriptional activity [21, 81–84], this phosphorylation is commonly used experimentally as evidence of STAT activation and proper function. STAT1, STAT2, and/or STAT3 tyrosine phosphorylation has been demonstrated by others to occur in fibroblast cells [85] or macrophages [40] at various times following infection with TC-MCMV. Others have recently demonstrated that 24 hrs following MCMV infection of fibroblasts, tyrosine phosphorylation of STAT1 and STAT3 does not necessarily confer transcriptional activation to these proteins, particularly during MCMV infection [85]. Although for the parameters of the present study the downstream functional activity of these phosphorylated STAT proteins remains to be seen, it is nonetheless possible that early MCMV-related SOCS1 and SOCS3 stimulation in IC-21 macrophages may be an indirect consequence of pSTAT1, pSTAT2, and/or pSTAT3 stimulation early during MCMV infection, possibly occurring through virally-mediated up-regulation of JAK/STAT-signaling cytokines. For this to be the case, it would mean either (i) the effects of MCMV infection on IFN and JAK/STAT signaling pathways under the parameters of this study are distinct from previous studies with TC-MCMV in fibroblasts or macrophages [37, 39, 40, 75, 85, 86], or (ii) that SOCS1 and/or SOCS3 are stimulated under the parameters of the current study by non-JAK/STAT signaling pathways or other virally induced mechanisms. Although JAK/STAT signaling is the major and best-studied induction pathway of SOCS proteins (reviewed in [27]), both SOCS1 and SOCS3 have been shown to undergo transcriptional stimulation by cellular pathways other than JAK/STAT [23–26]. Blockage of STAT pathways therefore does not necessarily preclude the induction of SOCS1 and/or SOCS3 by other cellular or viral mechanisms, even at later times of infection.

Nevertheless, SOCS1 and SOCS3 proteins were stimulated in IC-21 cells at 3 hpi with SG-MCMV RM4503, prior to visual detection of EGFP in these cells at 48 hpi, Given that the frequency of SOCS1+ or SOCS3+ immunofluorescently-stained cells approaches nearly 100% in SG-MCMV-infected IC-21 cells without IE2-driven EGFP detection (Fig 8), it appears evident that SOCS1 and SOCS3 stimulation during MCMV infection of IC-21 mouse macrophages occurs in uninfected bystander cells. Taken together with the transcriptional stimulation of SOCS-inducing cytokines such as IFN-γ and IL-6, these data suggest that SOCS1 and/or SOCS3 are indirectly stimulated in a paracrine fashion by virus-induced soluble factors and/or cellular pathways.

We tested the dispensability of MCMV DNA replication and/or late gene expression on MCMV-stimulated SOCS1 or SOCS3 production in MCMV-infected IC-21 mouse macrophages at 72 hpi by assessing the sensitivity of SOCS1 or SOCS3 expression to increasing doses of GCV, which inhibits HCMV and MCMV replication and subsequent expression of late viral genes [49]. That SOCS3, but not SOCS1, is sensitive to GCV treatment suggests divergent or temporally-driven mechanisms for stimulation of these proteins during late infection with MCMV. Furthermore, we found that SOCS3 mRNA is stimulated early during MCMV infection (2–6 hpi), but not at 24 hpi, and is then stimulated again at 72 hpi, suggesting a biphasic pattern of SOCS3 expression during MCMV infection of IC-21 macrophages. We have not eliminated the possibility, however, that this later stimulation of SOCS3 may be attributed to a second round of infection.

Taken together, the results of this study suggest that: (i) the virologic mechanism(s) of SOCS1 or SOCS3 expression during MCMV infection depends on cell type and virus passage origin, (ii) although neither MCMV IE1 nor IE2 is likely to govern SOCS1 and/or SOCS3 stimulation during SG-MCMV infection of IC-21 macrophages, a putative role remains for MCMV IE3, MCMV E, and/or tegument-packaged host or viral genetic material, (iii) direct SG-MCMV infection is not required to stimulate SOCS1 and SOCS3 expression in uninfected bystander macrophages, implicating a possible role for SOCS-inducing cytokines such as IFN γ or IL 6, and (iv) late-stage stimulation of SOCS3, but not SOCS1, during SG-MCMV infection of macrophages is biphasic and sensitive to GCV treatment.
